# Dietary Exposure of the Red-Crowned Crane (Grus japonensis) to Total and Methyl Mercury in Zhalong Wetland, Northeastern China

**DOI:** 10.1007/s12011-014-9993-8

**Published:** 2014-05-03

**Authors:** Jinming Luo, Yajie Ye, Yongjie Wang

**Affiliations:** Department of Science, Qiqihar University, 161006 Qiqihar, People’s Republic of China

**Keywords:** Habitat of red-crowned crane, Reed, Aquatic animal, Mercury contamination

## Abstract

To determine the dietary exposure of the migratory red-crowned crane to mercury (Hg), this study analyzed the concentrations of total mercury (T-Hg) and methyl mercury (MeHg) in its prey, i.e., reeds and three aquatic animal families (*Perccottus glenni* Dybowski, *Cybister japonicus* Sharp, and Viviparidae) in northeastern China. Results indicated that the Hg concentration in Zhalong Wetland was elevated through the food chain, and the prey of the red-crowned crane contained measurable levels of T-Hg and MeHg. In prey tissues, MeHg was the main form of the Hg element and accounted for 61 % of total Hg concentration in Viviparidae, 58 % in *C. japonicus* Sharp, and 85 % in *P. glenni* Dybowski. The highest T-Hg and MeHg concentrations ranged from 1.66 to 3.89 ppm and from 1.12 to 2.67 ppm, respectively, and they were detected in the feathers of the red-crowned cranes. The lowest T-Hg concentration was determined in the excretions of wild red-crowned cranes at 0.21 ppm; furthermore, the content of MeHg was below the detection limit. In Zhalong Wetland, the level of dietary exposure of the population of red-crowned cranes to Hg is below the threshold of Hg toxicity. Moreover, eggshells are suitable indicators of Hg risk levels to the red-crowned crane.

## Introduction

Mercury (Hg) is a relatively common element in the environment and comes in various organic and inorganic forms. However, it is a primary concern for water fowls because it affects their health [1]. Hence, Hg is undesirable and potentially hazardous to the environment and its biota [2]. The effect of Hg on aquatic environments has been investigated extensively [3–5]. According to these studies, large quantities of the Hg that enter in these environments are often attached to particulate matter [6] and are readily taken up by plants [7]. As a result, elevated Hg is returned to consumers at the upper trophic level [8]. The high concentration of Hg in soil generally enhances the Hg content in plants and water animals [9, 10]. Therefore, we must determine the buildup of Hg concentration in aquatic birds and their prey to detect net change in ecological systems.

Recent research has shown that the body burden of metals in animals is proportional to the metal content in the environment [8, 11, 12]. Frequently, environmentally sensitive aquatic plants (i.e., reed) [13], fish [14], and water birds are often employed to indicate the pollution levels of a metal-contaminated region [15–17].

The red-crowned crane (*Grus japonensis*) is a rare international species that has been near extinction since 2000, as indicated in the Red List of Endangered Species generated by the International Union for Conservation of Nature and Natural Resources [18]. Worldwide, its population is very small at 2,750 mature individuals. Although the resident population in Japan remains stable [19], the migratory population in mainland Asia continually declines because of the loss and degradation of wetlands for agricultural and industrial development [20]. Red-crowned cranes are omnivores and typically feed on aquatic plants (e.g., reed roots and stems) and water animals (e.g., fish, shells, and aquatic insects). Thus, their dietary intake of Hg is significantly affected by their prey [3]. This Hg eventually accumulates in the bodies of the red-crowned cranes, given that they roost and nest in stable sites for years. Thus, they may be chronically exposed to contaminated habitats and prey [21].

Zhalong National Nature Reserve (Zhalong Wetland), in the downstream Wuyur catchments, northeastern China, is one of the largest habitant and breeding sites for migratory red-crowned crane. More than 30 petrochemical plants located in the midstream of the catchments and large area of arable land around the wetland may bring about some amount of Hg accumulation and high probability of contact of cranes with the toxic chemical. An adverse factor is that all rare species such as the red-crowned cranes in China are protected in legislation, and any intentional killing of those rare water fowls is prohibited. An alternative approach is to investigate Hg contamination level in their habitats [13] and to examine their external tissues (e.g., feather and egg) [15, 17, 22] and excretions [16]. In our previous research, feces were found to contain a measurable level of Cd and suitable as an indicator for body burden of Cd on red-crowned crane [23]. However, data are scarce on body burden and dietary exposure to Hg on the water bird in this region.

This research examines the levels of total mercury (T-Hg) and methyl mercury (MeHg) in the prey of the red-crowned crane to estimate the exposure levels of red-crowned cranes and to determine whether their feathers, excretions (mainly feces), and eggshells can indicate Hg contamination in the bodies of these birds. The present study is the first to investigate the accumulation of Hg in and the dietary exposure of red-crowned cranes to this element in northeastern China. The results from this research enrich our understanding of the ecological safety of the health of migratory red-crowned cranes in China.

## Materials and Methods

### Study Area

**Fig. 1:**
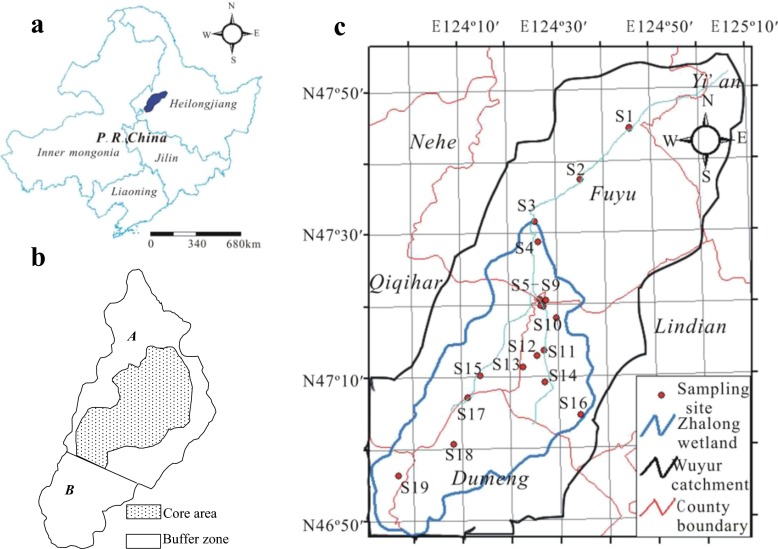
Location of Wuyur catchment (**a**) and infield sampling design (**b**, **c**)

The Wuyur River originates from the western foot of Xiaoxin’an Mountain, northeastern China, of which the watershed is presented to be an elongated strip and flows through the main food production zone of Heilongjiang Province in China (Fig. 1a), wherein contact with heavy metals like Hg is increased. The lower reaches of the river way are replaced by a large area of reed marsh after entering into the Zhalong Wetland. Zhalong Wetland covers an area of 2,100 km^2^ (123° 51′ to 124° 37′ E, 46° 48′ to 47° 32′ N) (core area, which is the roosting and breeding site of endangered water birds, e.g., red-crowned cranes, is approximately 700 km^2^; buffer zone occupies 1,400 km^2^ surrounding of the core area for protecting other common water birds) (Fig. 1b). A large area of pristine reed marsh attracts a population of more than approximately 500 migratory red-crowned cranes to inhabit and breed there from the late March to the early November (approximately 8 months) per year.

Tectonically, the wetland was formed by alluvial deposits with an average altitude of 140 m with 4,700 km^2^ of agricultural land around it (Fig. 1c). River feeding and precipitation are the major sources of water in this inland reed marsh. Climatically, the wetland has a typical temperate, semiarid, and semihumid continental monsoon climate with an average annual rainfall of 410 mm and a potential evaporation of 1,500 mm. The volume of runoff from upper reaches was abruptly decreased from 7.5 × 10^8^ m^3^ per year in the 1980s to less than 1 × 10^8^ m^3^ per year in the twenty-first century. Various sludges and wasted water from the surrounding residential area, agricultural land, and industrial workshop containing several types of toxic contaminants, like Hg, were discharged directly into the wetland without complete disposal treatment. The wastewater discharge volume increased from 0.17 × 10^8^ m^3^ in 1993 to 0.45 × 10^8^ m^3^ in 2010. Increasing amount of pollution discharges elevated various toxic element concentrations not only in the sediment but also in the entire biota.

### Sampling Scheme

A total of 19 sampling sites were designed for aquatic plant (i.e., reed) and aquatic animal collection. The first set of two sample sites (i.e., S1 and S2) was in the middle reaches of Wuyur catchments, the second set of ten sample sites (S3–S10, S15, and S16) was in buffer zone A of the Zhalong marsh, the third set of six sample sites (i.e., S11–S14, S17, and S18) was in the core area of the wetland, and the remaining one sample (S19) was designed in buffer zone B of the wetland (Fig. 1c). Three parts of reed organ, i.e., root, rhizome, and stem, and three aquatic animal families, i.e., *Perccottus glenni* Dybowski, *Cybister japonicus* Sharp, and Viviparidae, that are typical preys of the red-crown crane in the wetland were collected at designed sampling sites (see details in Table 1). Surface sediment at each site was sampled simultaneously. All of the prey samples were rinsed thoroughly in field with distilled water to remove pollutants attached on their body, then placed in car refrigerator at −4 °C, and transported back to the laboratory.

**Table 1:** Summary of numbers (Nums.) and average fresh weight (A.F.Wt.) in the three aquatic animal species sampled in the field

Species		S1	S2	S3	S4	S5	S6	S7	S8	S9	S10	S11	S12	S13	S14	S15	S16	S17	S18	S19
*Viviparidae*	Nums.	4	3	7	5	7	5	9	5	5	8	5	6	5	5	5	–	3	7	6
	A.F.Wt.	2.36	2.06	3.29	1.29	3.59	0.89	0.56	0.94	1.42	0.43	0.89	1.02	1.37	1.01	0.74	–	0.52	0.81	0.75
*C. japonicus* Sharp	Nums.	1	1	2	1	2	2	2	2	2	2	3	2	1	2	3	1	3	2	2
	A.F.Wt.	2.34	2.41	2.65	1.95	3.03	2.78	2.35	3.19	2.12	3.02	1.97	2.36	2.16	3.03	3.41	2.67	2.35	2.43	2.15
*P. glenni* Dybowski	Nums.	2	2	3	5	3	10	11	6	3	18	4	7	20	12	4	2	6	7	3
	A.F.Wt.	3.46	3.27	7.07	4.89	12.53	5.58	1.32	0.76	8.48	5.89	6.58	6.15	1.56	4.36	3.15	4.36	5.43	4.63	4.26

Excretions (mainly feces) of the red-crowned cranes were collected in late October of 2011, and after-hatch residual eggshells were collected from the field in late April and early May of 2012. The outer parts of feces were carefully removed, and the core parts of the feces were picked up to avoid the influence by the sediment. We collected residual eggshell samples left by the red-crowned cranes at four nesting sites (S10, S13, S15, and S17) and excretion at sites S15 and S17. Similarly, the eggshells were washed with distilled water in the field and immediately transferred to the laboratory.

Four red-crowned crane carcasses were collected at four nesting sites: one adult crane was collected in early April of 2010 at site S10 [body weight (BW) 9.4 kg, male]; two subadult cranes were found in early November at site S13 (BW 6.5 kg, female) and site S15 (BW 6.8 kg, male), and both dead cranes had almost no residues in their stomach, but with few grass seeds and stems; and one adult crane sample found in late October of 2012 at S17 (an obvious fracture injury was found in the right wing; BW 8.3 kg, male). The direct death cause for these crane samples was starvation because of food shortage in freezing condition as reported by our previous research [24]. The cranes were immediately transferred to the laboratory for dissection. Approximately, 1- to 2-g samples of livers, kidney, and breast muscles were collected using a stainless steel knife. Polyethylene gloves were used throughout all dissection procedures to prevent contamination. Some flight feathers were also collected from the cranes and washed with distilled water in the laboratory.

### Microwave Digestion and Element Analysis

After drying the reed organs, aquatic animal body, eggshell, and feathers of red-crowned crane samples with filter papers, these samples were oven-dried to a constant weight (48 h at 60 °C). The dried samples were ground to homogenous powders in a quartz bowl for acid digestion. Similar processes were performed on the feces subsamples, liver, kidney, and muscle of red-crowned crane, without washing and drying in the laboratory.

A total of 0.5 g of each category sample was acid digested in a microwave according to USEPA (1996) methods [25]. Five milliliters of 3:7 H_2_SO_4_:HNO_3_ (trace metal grade) was added to each subsample [26], which was then refluxed at about 150 °C for 3 h. Triplicate subsamples of known dry weight were digested in acid mixture, evaporated slowly to almost dryness (90 °C), and the residue was dissolved in 5 ml 1:1 diluted HCl and then settled to 25 ml for analysis after the solution cooled down to room temperature.

We determined T-Hg concentration in sediments, prey tissues, and the body and excretions of a red-crowned crane using a mercury analyzer (Tekran 2600 CVAFS; Tekran Instrument Corporation, Knoxville, USA) with a detection limit of 0.005 μg kg^−1^. Prior to Hg measurement, we used a reagent blank composed of 30 % HCl to set a zero reading. To verify the performance of the instrument, a certified reference material was analyzed before, during, and at the end of every run.

MeHg was extracted from the tissues of water animals and red-crowned cranes according to the method developed by Zheng et al. [27]. These tissues were dried in their digestion vessels. All of the subsamples were then heated overnight at approximately 60 °C prior to the addition of 50 ml of 2 M HCl and 1 ml of 1 % CuSO_4_ solution. After shaking for 10 min, the solution was filtered. The samples were buffered to pH 4 with either 2 M HCl or 6 M NaOH solution. Thereafter, the filtrate was passed through a sulfhydryl cotton tube filled with 0.1 g cotton and was eluted by 1 ml of 2 M HCl solution. This process was repeated twice more under additional solvent (1 ml). MeHg was extracted from the elution with benzene. It was then analyzed by gas chromatography with electron capture detection and a detection limit of 0.1 μg kg^−1^.

We estimated the precision and accuracy of the applied analytical method based on certified reference materials (GBW-07601 for T-Hg, 0.36 ± 0.05 mg kg^−1^; GBW-07604 for MeHg, 0.026 ± 0.003 mg kg^−1^) in relation to the measured values (trace metals in fish muscle and the feathers of the red-crowned crane). The results agreed with the certified values for all metals, with average recovery rates of 91.7 and 92.4 % for T-Hg and MeHg, respectively. All of the materials used for sampling and analysis were acid-washed. Moreover, all of the samples were analyzed in triplicate at a relative standard deviation lower than 1.5 %.

### Statistical Analysis

SPSS 10.0 for Windows was used for data analysis. Analysis of variance (ANOVA) was employed to test whether Hg concentrations varied significant between the reed organs and water animals in the buffer zone and core area. Possibilities less than 0.05 (*p* < 0.05) were considered statistically significant, and Pearson’s correlation coefficients were used to calculate correlations between the T-Hg and MeHg in the tissues of three aquatic animals and samples of red-crowned crane.

## Results

### Hg Contaminations in the Prey

In the sediments, Hg contents generally exceeded the upper limit of the natural background values (120 ppb) and ranged from 90 to 410 ppb (Table 2), with the exception of site S14. In the buffer zone of Zhalong Wetland, almost all of the Hg surpassed the Hg range (20 to 290 ppb) in China as reported by Kabata-Pendias [28]. At site S16, Hg concentration was extremely high at 410 ppb. This value exceeds the tolerable level (400 ppb) for agro-economic crops as provided by Kabata-Pendias [28].

**Table 2:** Concentrations of T-Hg and MeHg in sediment, three organ parts of reed, and three water animal species in Wuyur River (ppb in dry weight, *n* = 3)

	Sediment	Reed			Viviparidae		*C. japonicus* Sharp		*P. glenni* Dybowski	
		Root	Rhizome	Stem	T-Hg	MeMg	T-Hg	MeMg	T-Hg	MeMg
S1	290	46.78	12.72	6.14	56.23	32.38	66.47	33.94	80.45	72.90
S2	360	88.67	24.54	10.62	95.48	54.85	128.49	72.02	156.47	112.96
S3	350	61.35	18.55	9.64	85.16	41.95	135.76	62.45	135.47	100.32
S4	310	76.37	36.53	7.39	79.88	26.11	118.48	63.86	125.37	96.26
S5	320	70.63	33.67	8.14	82.31	39.86	116.78	68.08	108.12	88.72
S6	410	86.7	40.25	11.21	112.04	48.53	153.154	109.83	122.74	107.56
S7	340	23.97	10.48	8.38	102.36	47.42	120.89	73.73	112.12	84.90
S8	330	31.39	10.78	7.64	79.56	30.51	102.36	82.84	90.24	74.30
S9	375	43.78	15.56	9.88	110.45	40.02	138.15	76.35	133.45	90.46
S10	310	35.48	12.6	7.14	104.65	43.07	208.34	100.39	182.19	148.74
S11	170	19.48	6.45	4.89	56.78	30.65	68.45	33.95	92.45	67.70
S12	140	11.12	5.67	3.01	50.47	22.11	65.215	32.96	76.45	45.56
S13	160	15.63	6.487	3.84	39.59	17.85	42.02	28.93	40.16	27.02
S14	90	24.57	9.43	5.39	28.45	12.31	46.27	18.29	39.87	20.48
S15	310	33.87	11.27	6.88	103.27	46.11	133.47	53.94	112.47	90.774
S16	390	54.47	24.05	7.13	138.14	84.78	189.09	130.90	143.02	126.91
S17	230	35.46	27.86	4.85	108.36	82.09	120.36	66.57	114.45	102.64
S18	210	20.78	9.67	5.64	89.45	30.48	99.23	34.39	102.12	40.78
S19	260	48.89	18.76	6.88	99.09	60.07	156.25	72.04	132.36	74.34

Reed, as the preferred food of the red-crowned cranes in Zhalong wetland, was found to contain measurable levels of Hg, with the following order of increasing concentration: stem < rhizome < root (Table 2). The T-Hg concentrations in the reed root and rhizome varied from 11.12 to 88.67 ppb and from 5.67 to 40.25 ppb. The contents of T-Hg in reed root were correlated with the concentrations in the sediment at the significant level of 0.01 (*r* = 0.78, *p* < 0.001). T-Hg contents in sediments and reed organs of the buffer zone were significantly larger than these in the core area (*F* = 17.29, *p* < 0.001, for sediment; *F* = 10.33, *p* = 0.005, for reed root; *F* = 6.56, *p* = 0.04, for rhizome; and *F* = 20.56, *p* < 0.001, for reed stem).

**Fig. 2:**
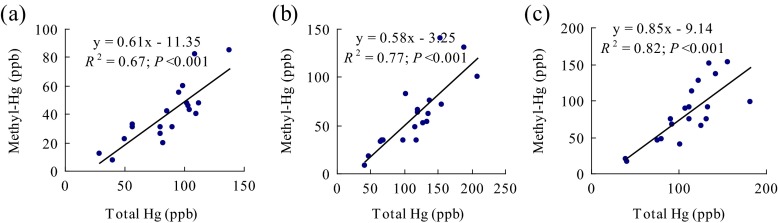
Relation between the concentrations (dry weight) of total mercury and methyl mercury in three aquatic animals (**a** Viviparidae, **b** *C. japonicus* Sharp, **c** *P. glenni* Dybowski)

Hg was detected in the tissues of three aquatic animals. This Hg mainly came in the form of MeHg (61 % in Viviparidae, 58 % in *C. japonicus* Sharp, and 85 % in *P. glenni* Dybowski) (Table 2, Fig. 2). The Viviparidae family is the primary consumer and prefers to feed on reed leaves and humus in the sediments. This species accumulates relatively low concentrations of T-Hg and MeHg (28.45 to 138.14 ppb and 12.31 to 84.78 ppb, respectively). By contrast, the aquatic animals in the high trophic level of the food chain (i.e., the secondary consumers *P. glenni* Dybowski and *C. japonicus* Sharp families, which mainly prey on fish and the larvae of aquatic insects) had higher concentrations (42.02 to 208.34 ppb and 18.29 to 130.90 ppb of T-Hg and MeHg, respectively, in *C. japonicus* Sharp; 39.87 to 182.19 ppb and 20.48 to 148.74 ppb of T-Hg and MeHg, respectively, in *P. glenni* Dybowski).

### Hg in the Red-Crowned Cranes

The red-crowned cranes accumulated measurable levels of Hg. The order of contamination is as follows: feces < muscle < eggshell < liver < kidney < feathers (Table 3). The feathers contained the highest concentrations of T-Hg and MeHg at 3.89 and 2.67 ppm, respectively. Furthermore, the Hg concentration in the internal organs was relatively high. In the kidney, T-Hg content varied from 0.89 to 2.73 ppm, and MeHg concentration ranged from 0.62 to 2.46 ppm. In the liver, T-Hg varied from 0.68 to 2.48 ppm, and MeHg concentration ranged from 0.48 to 2.24 ppm. Furthermore, these organs were the most prone to Hg accumulation, i.e., the 2.73 ppm of T-Hg accumulated in the kidney was below the sublethal level (3 to 13 ppm) recommended by Eisler [3]. The feces had the lowest T-Hg concentration at 0.18 to 0.23 ppm. Moreover, the MeHg concentrations were close to the analytical detection limits. The muscles also possessed low metal concentrations. In the eggshells obtained from sites S10 and S13, the T-Hg concentrations were 1.39 and 1.16 ppm, respectively, which were above the sublethal level (1 ppm) of Hg content in bird eggs according to Burger and Gochfeld [22]. In addition, T-Hg concentration is significantly lower in the migratory red-crowned cranes of China than in the resident cranes of Japan (*F* = 37.78, *p* < 0.001).

**Table 3:** Hg (T-Hg/MeHg) concentration (ppm) in the flight feathers, feces, internal organs (liver and kidney), and muscles of red-crowned cranes from the study area (means, *n* = 3, dry weight) and comparison of Hg enrichment level with non-migratory red-crowned crane in Japan

Species	Sampling site	Plume	Feces	Eggshell	Liver	Kidney	Muscle
Migratory crane in China	S10	3.89 (2.64–4.85)/2.67 (1.87–3.28)	–	1.39 (0.74–1.55)/1.02 (0.66–1.48)	2.48 (1.56–3.34)/2.24 (1.42–2.96)	2.73 (1.36–3.94)/2.46 (1.22–3.67)	0.58 (0.26–0.70)/0.38 (0.18–0.46)
	S13	2.85 (2.47–3.26)/1.89 (1.53–2.48)	–	1.16 (0.42–1.36)/0.87 (0.38–1.22)	1.84 (0.59–2.64)/1.46 (0.43–2.21)	2.12 (1.62–2.86)/1.68 (1.31–2.29)	0.32 (0.16–0.39)/0.21 (0.09–0.32)
	S15	1.66 (1.23–2.15)/1.12 (0.86–1.53)	0.18 (0.08–0.29)/ND	0.89 (0.68–1.18)/0.56 (0.45–0.79)	1.57 (1.02–2.24)/1.25 (0.81–1.83)	1.94 (1.52–2.97)/1.47 (1.34–2.55)	0.24 (0.11–0.28)/0.16 (0.07–0.21)
	S17	1.68 (1.30–2.21)/1.18 (0.78–1.67)	0.23 (0.11–0.33)/ND	0.58 (0.34–0.86)/0.39 (0.21–0.65)	0.68 (0.43–0.92)/0.48 (0.34–0.68)	0.89 (0.42–1.33)/0.62 (0.33–1.15)	0.20 (0.12–0.28)/0.13 (0.07–0.18)
	Average	2.51 (1.23–4.85)/1.75 (0.78–3.28)	0.21 (0.08–0.33)/ND	1.05 (0.34–1.55)/0.71 (0.21–1.48)	1.64 (0.43–3.34)/1.36 (0.34–2.96)	2.04 (0.42–3.94)/1.56 (0.33–3.67)	0.34 (0.11–0.70)/0.18 (0.07–0.46)
Island crane in Japan [19]	Hokkaido (adult/male)	8.20 (1.00–24.10)	–	–	22.32 (ND–142.09)	41.98 (0.83–343.63)	2.35 (0.01–10.21)
	Hokkaido (adult/female)		–	–	11.9 (0.5–44.30)	26.73 (1.11–38.63)	1.38 (ND–9.48)
	Hokkaido (subadult/female)	3.6 (0.60–14.00)	–	–	5.77 (1.11–21.88)	4.38 (0.72–12.66)	0.03 (ND–0.09)

**Fig. 3:**
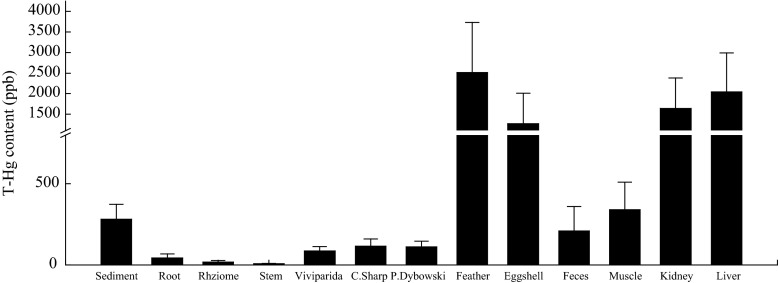
Average content ± SD of T-Hg in sediments, reed stems, three water animal families, and red-crowned crane (*n* = 3, dry weight)

The T-Hg and MeHg concentrations in the eggshells were significantly correlated with those in the liver and kidney (*r* = 0.985, *p* < 0.001, for eggshell vs. liver; *r* = 0.967, *p* < 0.001, for eggshell vs. kidney). However, these concentrations were not significantly correlated with those in the muscle (Table 4). Nonetheless, T-Hg and MeHg in the muscle were significantly correlated with those in the feathers (*r* = 0.960, *p* < 0.001). In the core area (main habitat of the red-crowned crane), the daily intake of Hg by these cranes ranged from 39.94 to 48.03 ppb, whereas the intake ranged from 54.39 to 65.09 ppb in the buffer zone (breeding and foraging sites for other protected water birds) (Table 5). Based on the average concentrations, T-Hg and MeHg were bioconcentrated and biomagnified through the food chain in Zhalong Wetland, as presented in Fig. 3.

**Table 4:** Correlation of T-Hg and MeHg in the feather and three internal organs of red-crowned crane

	Plume	Eggshell	Liver	Kidney	Muscle
Eggshell	0.923/0.936	1			
Liver	0.870/0.915	0.985^a^/0.942	1		
Kidney	0.880/0.872	0.967^a^/0.933	0.992^b^/0.993^b^	1	
Muscle	0.960^a^/0.965^a^	0.896/0.893	0.886/0.960^a^	0.925/0.919	1

^a^Significant at 0.05 level (two-tailed)

^b^Significant at 0.01 level (two-tailed)

**Table 5:** Estimated intake rates of Hg for the migratory red-crowned crane population using the five major types of their food

Item	Food consumed (g day^−1^)	Contents (ppb)	
		Buffer zone	Core area
Reed stem	110–130	8.46	5.17
Reed rhizome	140–160	21.31	12.61
Viviparidae	100–140	91.56	76.81
*P. glenni* Dybowski	120–140	125.36	90.12
*C. japonicus* Sharp	200–230	131.37	95.54
Total	670–770 [35]	54.39–65.09	39.94–48.03

## Discussions

The present study reveals two little known adverse effects of Hg (primarily MeHg) on the bodies of red-crowned cranes in China’s Zhalong Wetland. First, the prey of these cranes contained measurable levels of T-Hg and MeHg, but the dietary exposure level of this population to Hg was below the Hg toxicity threshold. Second, the Hg concentration levels in the feathers and eggshells of these cranes were significantly high and were correlated with those in their internal organs (i.e., liver, kidneys, and muscles). However, the Hg content in the feces of the water bird was either low or even below the detection limit.

The finding of this study agreed with the conclusion drawn by Eisler [3] with respect to the bioaccumulation of Hg in the habitat of the red-crowned crane. According to Eisler [3], Hg is not only bioconcentrated by organisms but also biomagnified through the food chain. Barr [29] determined that loons laid fewer eggs when the Hg concentrations in their prey averaged 300 to 400 ppb. Furthermore, they did not lay eggs when this average exceeded 400 ppb. In the current study, the Hg contents in all of the prey did not exceed the allowable concentration limit (200 ppb) recommended by Agah et al. [30] and the toxicity threshold concentration (300 ppb) provided by Barr [29]. This result suggests that the incidence of Hg in prey does not contribute highly to the diets of water birds (such as red-crowned cranes and other water fowls) in this region.

The present study reveals that MeHg dominates the Hg content in prey tissues and in the bodies of water birds. This finding is consistent with those of many previous researches. According to Burger and Gochfeld [22] and Evers et al. [31], muscle tissue provides the protein used to form feathers. Furthermore, the feathers are among the few body compartments in which MeHg can be remobilized. Therefore, the Hg concentrations in feathers during growth can indicate MeHg contamination in the body of the red-crowned crane. In the current study, the T-Hg concentrations in the feathers of the red-crowned crane in Zhalong Wetland were similar to the average Hg concentrations in the Little Egrets of Tai Lake (0.64 ppm) and Poyang Lake (0.41 ppm) in Southern China as well as those of Common Eiders in Aleutian Island (0.98 ppm) and in the Amchitak and Kiska area (0.64 ppm) as reported by Zhang et al. [4] and Burger et al. [8]. However, the concentrations determined by the present study were lower than those in the Little Egrets of Pearl Delta (2.09 ppm) (*p* < 0.05) in China.

In the current study, the increasing Hg content in the feathers of red-crowned cranes demonstrated that crane feathers are prone to Hg contamination, as with the feathers of many other birds [8, 22]. The T-Hg and MeHg concentrations in the feathers of the red-crowned crane exceed those in their internal organ tissues (liver and kidney). This finding contradicts those of many previous researches. This discrepancy is most likely influenced by external contamination (i.e., atmospheric deposition) because exogenous fractions of heavy metals cannot be completely removed by washing alone [17]. Therefore, the use of crane feathers as indicators of Hg pollution may be challenged, and further research is recommended.

The T-Hg and MeHg concentrations in the eggshells are strongly correlated with those in the liver and kidney. However, they are not significantly correlated with the concentrations in the muscle. This result agrees with that of Evers et al. [31], which postulates that MeHg in the muscle contributes only slightly to Hg concentration in the developing egg. Burger et al. [8] reported that the T-Hg content in eggs normally ranges between 10 and 20 % of that in the liver. In the present study, the eggshells of the red-crowned crane removed Hg from its body, as indicated by the relatively high T-Hg and MeHg concentrations therein. The eggshells of birds such as the Great Tit (*Parus major*) [16] and common loon [5, 16] perform a similar function. Given that Chinese law prohibits the intentional killing of wild cranes and the picking of their eggs, the eggshells of these birds can effectively indicate the Hg risk level to the red-crowned crane.

Our results showed that the T-Hg concentrations in feces were low and that MeHg concentrations therein were below the analytical detection limit. Dauwe et al. [16] determined that the Great Tit could excrete Hg from its body. Hence, its feces can indicate the pollution levels in a metal-contaminated region. Conversely, Leonzio et al. [32] reported that the total Hg and MeHg concentrations in feces are limited. Our results confirm this finding because the feces of the red-crowned crane do not always reflect the contamination status of Hg in its body. Thus, excretions may not be a suitable indicator of Hg risk level.

Similar to the results reported by Takazawa et al. [33] and Malik et al. [34], we determined relatively high Hg contents in the kidney and liver tissues of the red-crowned crane. According to these previous studies, the livers and kidneys of water fowls are prone than other internal organs to the accumulation of many toxic metals, such as Hg. According to Eisler [3], Hg concentrations that exceed 3 ppm in kidney or liver tissue are presumptive evidence of an environmental Hg problem. In the present study, however, the T-Hg concentrations in the internal organs of the red-crowned crane were below the concentration threshold of Hg toxicity, thereby suggesting that the Hg risk level to the red-crowned crane was low.

The daily Hg intake of the red-crowned crane (39.94 to 48.03 ppb) was significantly lower than the concentration at the sublethal effect level recommended by Eisler [3] for sensitive aquatic bird species (i.e., 100 to 500 ppb day^−1^). Hence, we regard the level of dietary exposure of the migratory red-crowned cranes to Hg as safe in China. However, the elevated Hg levels in the buffer zone of Zhalong Wetland and the detectable levels of T-Hg and MeHg in red-crowned cranes should be examined further.
